# Inflammasome Sensor Nlrp1b-Dependent Resistance to Anthrax Is Mediated by Caspase-1, IL-1 Signaling and Neutrophil Recruitment

**DOI:** 10.1371/journal.ppat.1001222

**Published:** 2010-12-09

**Authors:** Mahtab Moayeri, Devorah Crown, Zachary L. Newman, Shu Okugawa, Michael Eckhaus, Christophe Cataisson, Shihui Liu, Inka Sastalla, Stephen H. Leppla

**Affiliations:** 1 Laboratory of Bacterial Diseases, Bacterial Toxins and Therapeutics Section, National Institute of Allergy and Infectious Diseases, National Institutes of Health, Bethesda, Maryland, United States of America; 2 Diagnostic and Research Services Branch, Division of Veterinary Resources, Office of Research Services, National Institutes of Health, Bethesda, Maryland, United States of America; 3 Laboratory of Cancer Biology and Genetics, Center for Cancer Research, National Cancer Institute, National Institutes of Health, Bethesda, Maryland, United States of America; University of New Mexico, United States of America

## Abstract

*Bacillus anthracis* infects hosts as a spore, germinates, and disseminates in its vegetative form. Production of anthrax lethal and edema toxins following bacterial outgrowth results in host death. Macrophages of inbred mouse strains are either sensitive or resistant to lethal toxin depending on whether they express the lethal toxin responsive or non-responsive alleles of the inflammasome sensor Nlrp1b (*Nlrp1b^S/S^* or *Nlrp1b^R/R^*, respectively). In this study, Nlrp1b was shown to affect mouse susceptibility to infection. Inbred and congenic mice harboring macrophage-sensitizing *Nlrp1b^S/S^* alleles (which allow activation of caspase-1 and IL-1β release in response to anthrax lethal toxin challenge) effectively controlled bacterial growth and dissemination when compared to mice having *Nlrp1b^R/R^* alleles (which cannot activate caspase-1 in response to toxin). Nlrp1b^S^-mediated resistance to infection was not dependent on the route of infection and was observed when bacteria were introduced by either subcutaneous or intravenous routes. Resistance did not occur through alterations in spore germination, as vegetative bacteria were also killed in *Nlrp1b^S/S^* mice. Resistance to infection required the actions of both caspase-1 and IL-1β as *Nlrp1b^S/S^* mice deleted of caspase-1 or the IL-1 receptor, or treated with the Il-1 receptor antagonist anakinra, were sensitized to infection. Comparison of circulating neutrophil levels and IL-1β responses in *Nlrp1b^S/S^,Nlrp1b^R/^* ^R^ and IL-1 receptor knockout mice implicated Nlrp1b and IL-1 signaling in control of neutrophil responses to anthrax infection. Neutrophil depletion experiments verified the importance of this cell type in resistance to *B. anthracis* infection. These data confirm an inverse relationship between murine macrophage sensitivity to lethal toxin and mouse susceptibility to spore infection, and establish roles for Nlrp1b^S^, caspase-1, and IL-1β in countering anthrax infection.

## Author Summary

In this study, we show that anthrax lethal toxin activation of Nlrp1b in toxin-sensitive mouse macrophages imparts resistance to infection. Inbred and congenic mice harboring macrophage-sensitizing Nlrp1b alleles control bacterial growth and dissemination independent of infection route or effects on germination efficiency. Knockout mice demonstrate that resistance imparted by Nlrp1b requires caspase-1 activity and IL-1 signaling. Mice in which lethal toxin activates the Nlrp1b inflammasome show an IL-1β response and increased neutrophil recruitment leading to increased resistance to infection. Neutrophil depletion experiments verify the importance of this cell type in resistance to *B. anthracis* infection. These data confirm an inverse relationship between murine macrophage sensitivity to lethal toxin and mouse susceptibility to spore infection and demonstrate that the activation of the inflammasome in response to anthrax infection in mice is a protective event that occurs through IL-1β induction of neutrophil recruitment.

## Introduction

Anthrax disease occurs following germination of *Bacillus anthracis* spores that enter an animal host through inhalational, oral, or cutaneous routes. Anthrax lethal toxin (LT), responsible for the lethality associated with terminal anthrax disease, is composed of two polypeptides. LF (lethal factor, a protease) is transported into the cytosol by PA (protective antigen, the cell-binding component). LF cleaves members of the mitogen-activated protein kinase kinase family (MEKs) [1]–[2]. LF's proteolytic activity is required for its ability to induce vascular collapse in animals and for the rapid lysis of macrophages and dendritic cells from certain inbred rodent strains (for review see [3]). The link between the cleavage of MEKs and LT's cytotoxic effects in macrophages and animals is currently unknown.

The genetic locus controlling the susceptibility of mouse and rat macrophages to LT has been mapped to *Nlrp1b,* a gene encoding a member of the NLR (Nod-like receptor) protein family [4], [5]. NLR proteins are the sensor components of inflammasome complexes which recruit and activate caspase-1 in response to intracellular danger signals [6], [7]. LT activates caspase-1 only in macrophages from mice harboring *Nlrp1b^S/S^* alleles, which express Nlrp1b^S^ proteins containing the polymorphisms required for LT responsiveness. Macrophages from mice harboring *Nlrp1b^R/R^* alleles and expressing Nlrp1b^R^ proteins are resistant to LT. Caspase-1 activation in Nlrp1b^S^ expressing macrophages is required for rapid LT-mediated lysis [4], [8], [9]. Earlier *in vivo* studies of LT-treated mice showed that rapid, transcription-independent IL-1β release occurs only in mouse strains having LT-sensitive macrophages (*Nlrp1b^S/S^*) [10], [11]. While the LT-induced activation of caspase-1, macrophage lysis, and IL-1β release in mice expressing Nlrp1b^S^ leads to a strong cytokine burst, this macrophage response is not consistently predictive of increased whole animal sensitivity to LT [10]. C3H/HeJ and C3H/HeOuJ mice, for example, harbor *Nlrp1b^S/S^* alleles but are resistant to toxin doses that kill some inbred strains having *Nlrp1b^R/R^* alleles [10].

LT also plays a role in the early stages of anthrax infection, prior to eliciting its systemic toxic effects. Bacterial dissemination and susceptibility studies using isogenic toxin component mutants [12] and anthrax toxin receptor knockout mice [13] provide evidence of LT's significant contribution to establishing infection. Some *in vitro* analyses suggest that toxin action is required for release of dividing intracellular bacteria from macrophages [14] or for spore survival against killing by macrophages [15]. In turn, while some reports suggest macrophages are required for the germination and transportation of spores from the site of infection to regional lymph nodes [14], [16], other investigations have indicated that the macrophage plays a protective role by killing spores [17]–[19].

We investigated the role of Nlrp1b in several anthrax infection models to form a better understanding of LT's role in establishing disease. Our data confirm an inverse relationship between murine macrophage sensitivity to LT and mouse susceptibility to spore infection. We demonstrate that the activation of the inflammasome in response to anthrax infection in mice is a protective event. Mice expressing Nlrp1b^S^ and harboring LT-sensitive macrophages were resistant to infection by both spores and vegetative bacteria at doses lethal to Nlrp1b^R^-expressing mice harboring LT-resistant macrophages. This Nlrp1b^S^-mediated resistance to *B. anthracis* infection required both caspase-1 and IL-1β mediated control of neutrophil responses. We propose a role for LT-mediated, Nlrp1b-dependent caspase-1 activation and IL-1β release in the control of *B. anthracis* infection.

## Results

### Susceptibility of inbred mouse strains to spore infection is controlled by *Nlrp1b*

**Figure 1:**
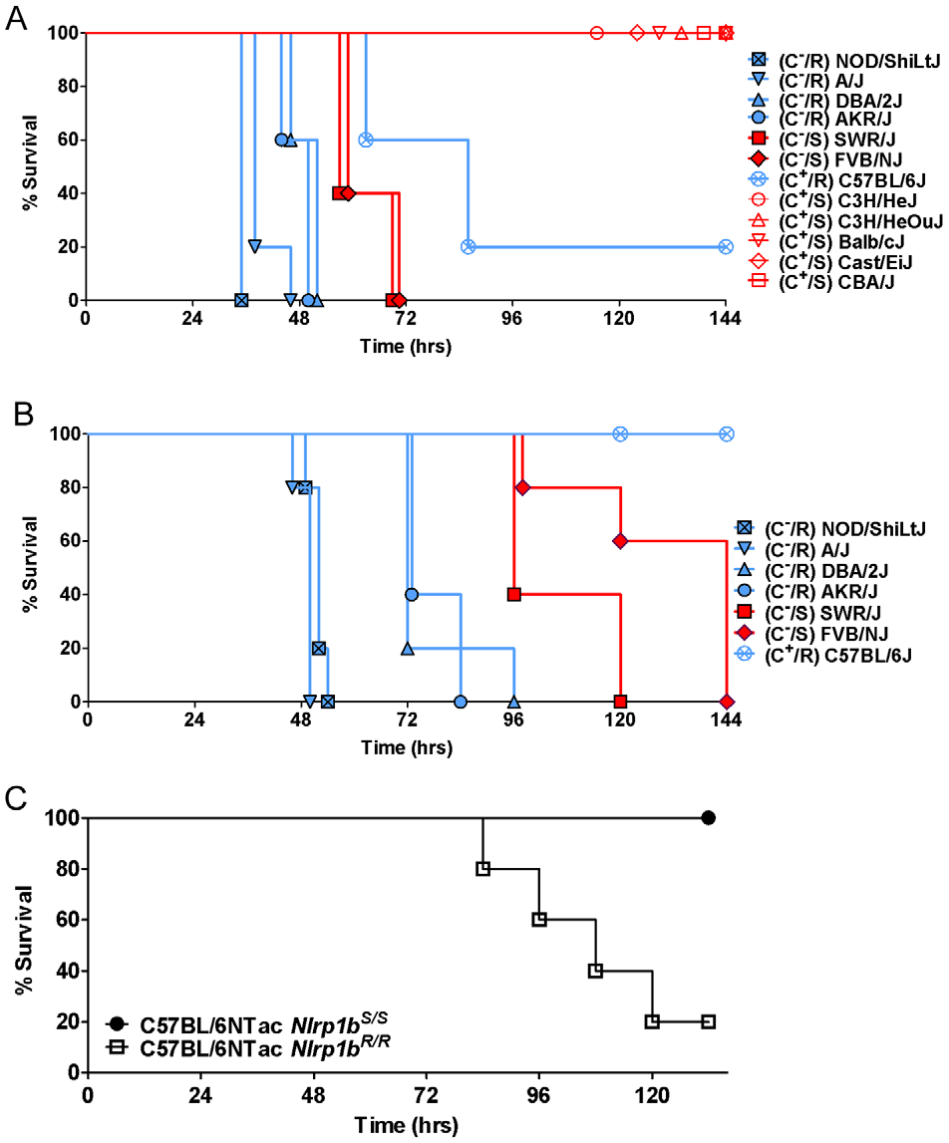
Susceptibility of inbred, C57BL/6NTac*Nlrp1b^S/S^* and C57BL/6NTac*Nlrp1b^R/R^* mouse strains to subcutaneous spore infection. Mice were injected SC with 2×10^6^ (A) or 2×10^5^ (B) spores and monitored for malaise. R indicates *Nlrp1b^R/R^* genotype and LT-resistant macrophages (plotted in blue). S indicates *Nlrp1b^S/S^* genotype and LT-sensitive macrophages (plotted in red). C^−^ and C^+^ indicate complement C5 status, with closed symbols used for C^−^ strains and open circles for C^+^ strains. For the representative experiments shown, n = 5 for all strains. Similar results were obtained in three independent experiments utilizing all strains except CBA/J, C3H/HeJ and C3H/HeOuJ, which were not used in subsequent studies. For panel A, logrank-based P-values comparing survival curves of any of the NOD/ShiLtJ, A/J, DBA/2J, and AKR/J mice (C^−^, *Nlrp1b^R/R^*) with the SWR/J and FVB/NJ (C^−^, *Nlrp1b^S/S^)* mice are <0.0035. P-values comparing the C57BL/6J mice (C^+^, *Nlrp1b^R/R^*) to any of C3H/HeJ, C3H/HeOuJ, Balb/cJ, Cast/EiJ, and CBA/J mice (C^+^, *Nlrp1b^S/S^*) are <0.015. All survival curves for C^+^ mice are significantly different than all survival curves for C^−^ mice (P-values <0.003) except C57BL/6J mice compared to FVB/NJ or SWR/J (P-value  = 0.063 for both comparisons). For panel B, P-values comparing survival curves of any of the NOD/ShiLtJ, A/J, and DBA/2J mice (C^−^, *Nlrp1b^R/R^*) with the SWR/J and FVB/NJ mice (C^−^, *Nlrp1b^S/S^*) are <0.004. Comparison of survival of C57BL/6J mice to all other mice except FVB/NJ yields P-values <0.0025. P-value for C57BL/6J compared to FVB/NJ survival is 0.0034. (C) C57BL/6NTac*Nlrp1b^S/S^* and C57BL/6NTac*Nlrp1b^R/R^* mice (n = 5/strain) were injected with 1×10^7^ spores and monitored. P-value (Logrank Test) comparing these survival curves is 0.0133. Similar results were obtained in three independent experiments, and in two studies using 2×10^7^ spores.

An initial survey of subcutaneous infection of 11 inbred mouse strains with the Ames 35 (A35) *B. anthracis* strain at four doses (1×10^8^, 2×10^7^, 2×10^6^ and 2×10^5^ spores/mouse) identified a range of sensitivities to infection (Fig. 1, and data not shown). Approximately half of the commonly studied inbred mouse strains are deficient in complement C5. We noted that all complement C5 component-deficient mice (C^−^) were fully susceptible to the lowest spore dose (2×10^5^). At doses below 1×10^8^, all complement-sufficient (C^+^) strains (Balb/cJ, Cast/EiJ, C3H/HeJ, C3H/HeOuJ) except one (C57BL/6J) were resistant to infection (Fig. 1A, 1B, and data not shown). The observation that complement sufficiency contributed to resistance to spore infection supported previous findings [20]–[22]. However, we found that within both the C^−^ and C^+^ groups, mice harboring *Nlrp1b^R/R^* alleles, and thus LT-resistant macrophages, were significantly more susceptible to spore infection than *Nlrp1b^S/S^* mice harboring LT-sensitive macrophages (Fig. 1A, 1B). Spore infections in congenic mice harboring *Nlrp1b^S/S^* or *Nlrp1b^R/R^* alleles on a C57BL/6NTac background confirmed that Nlrp1b^S^ expression conferred a higher degree of resistance to spore infection (Fig. 1C), supporting similar findings by other investigators using Nlrp1b^S^ transgenic mice [23]. Thus, Nlrp1b^S^ appears to manifest a protective effect independent of complement status.

### Nlrp1b controls *B. anthracis* clearance and dissemination

**Figure 2:**
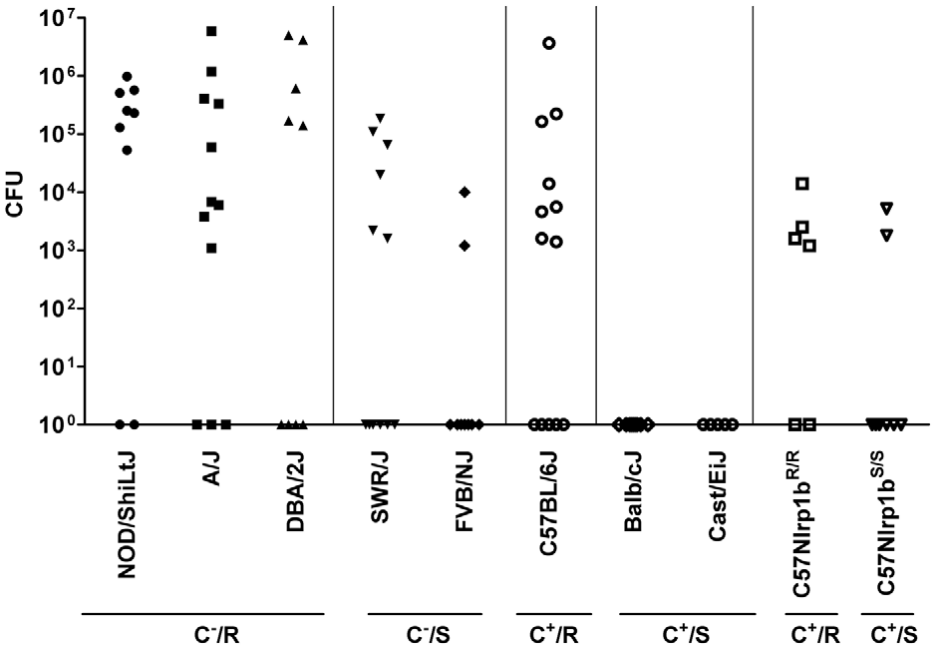
Bacterial CFU counts in organs following spore infection. Mice were injected with 2×10^7^ spores (SC) and spleen, liver and kidneys were harvested at 24–32 h, pooled, and the bacterial numbers (CFU/mouse) in the combined organs were assessed by dilution plating. C^+^, C^−^, R, and S designations are as in Fig. 1. Results represent three independent experiments and n values are n = 9 for all strains except A/J (n = 12), SWR/J (n = 12), C57BL/6J (n = 13), CAST/EiJ (n = 5), C57BL/6NTac *Nlrp1b^R/R^* (n = 6) and C57BL/6NTac *Nlrp1b^S/S^* (n = 8). F-test (which compares the variance between groups) yields values >0.05 comparing all groups except the following, which have significant variance (<0.05): NOD/ShiLtJ vs. SWR/J, FVB/NJ, C57BL/6J, Balb/cJ and Cast/EiJ, C57BL/6NTac *Nlrp1b^S/S^*, C57BL/6NTac *Nlrp1b^R/R^*; A/J vs. SWR/J, FVB/NJ, Balb/cJ and Cast/EiJ, C57BL/6NTac *Nlrp1b^S/S^*, C57BL/6NTac *Nlrp1b^R/R^*; DBA/2J vs. Balb/cJ and Cast/EiJ, C57BL/6NTac *Nlrp1b^S/S^*, C57BL/6NTac *Nlrp1b^R/R^*; SWR/J vs. every group; FVB/NJ vs. every group except C57BL/6NTac *Nlrp1b^S/S^* and C57BL/6NTac *Nlrp1b^R/R^*; C57BL/6J vs. every group except A/J and DBA/2J. Balb/cJ and Cast/EiJ compared to every group except C57BL/6NTac *Nlrp1b^S/S^*. The P-value comparing variance between C57BL/6NTac *Nlrp1b^S/S^* and C57BL/6NTac *Nlrp1b^R/R^* is 0.0145.

Analysis of bacterial dissemination 24–32 h after subcutaneous (SC) spore infection (2×10^7^/mouse) in 10 inbred strains showed that C^−^ strains expressing Nlrp1b^R^ (NOD/ShiLtJ, A/J, and DBA/2J) had higher bacterial counts in organs (pooled spleen, liver and kidney) than their C^−^ Nlrp1b^S^-expressing counterparts (SWR/J, FVB/NJ) (Fig. 2, see statistical comparisons for groups in legend). Similarly, C^+^ strains expressing Nlrp1b^R^ (C57BL/6J) had significantly higher counts than their C^+^ Nlrp1b^S^-expressing counterparts (Balb/cJ and Cast/EiJ). Thus, the *Nlrp1b^S^* allele appeared to inhibit control of bacterial spread and growth compared to the *Nlrp1b^R^* allele, irrespective of complement status. Similar results were obtained for dissemination to the lung (data not shown).

### Nlrp1b does not alter germination efficiency

**Figure 3:**
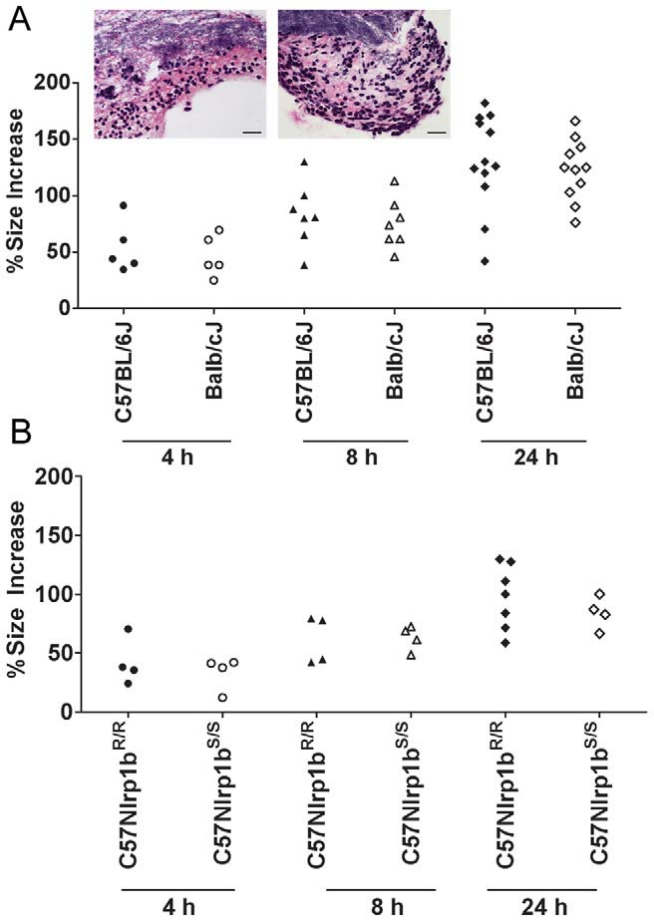
Nlrp1 does not control spore germination. Percent increases in foot edema dorsal/ventral measurements relative to PBS-treated controls are shown at 4, 8, and 24 h following spore injection (2×10^7^, 50 µl, SC). P-values comparing Balb/cJ and C57BL/6J at all time points were >0.388 (panel A). P-values comparing C57BL/6NTac *Nlrp1b^S/S^* and C57BL/6NTac *Nlrp1b^R/R^* at all three time points were >0.5 (panel B). Inset panels show H&E (or B&H) staining of skin sections in C57BL/6J (left) or Balb/cJ (right) mice 8 h after SC spore infection (2×10^7^). Germinated bacteria can be seen in the upper half of each panel with neutrophil influx in the lower half. Neutrophil influx was also verified by myeloperoxidase immunostaining (not shown). Image is taken at 400× magnification (scale bar  = 25 microns).

Nlrp1b^S^- and Nlrp1b^R^-expressing mice were compared for differences in their support of spore germination at the initial infection site by measuring edema toxin production (and resultant local edema), which can occur only after germination. Foreleg inoculation of 2×10^7^ spores in a small volume (50 µl) produced a local, restricted edematous lesion that could be quantified by measuring its anterior to posterior and sagittal dorsal/ventral dimensions. Edema was observed in all strains as early as 1–2 h following infection (data not shown), and the sizes of the lesions in Nlrp1b^S^- and Nlrp1b^R^-expressing strains were not significantly different over 4–24 h (Fig. 3). Histological analyses of skin sections verified an almost complete germination of spores within hours of infection (Fig. 3, inset), in a manner that did not appear to require uptake by macrophages or dendritic cells, which were not yet present in these sites. Thus, bacterial germination at the injection site and resulting edema toxin production occur independent of Nlrp1b status.

### Nlrp1b^S^ mediates resistance to vegetative bacilli in a manner independent of the route of infection

**Figure 4:**
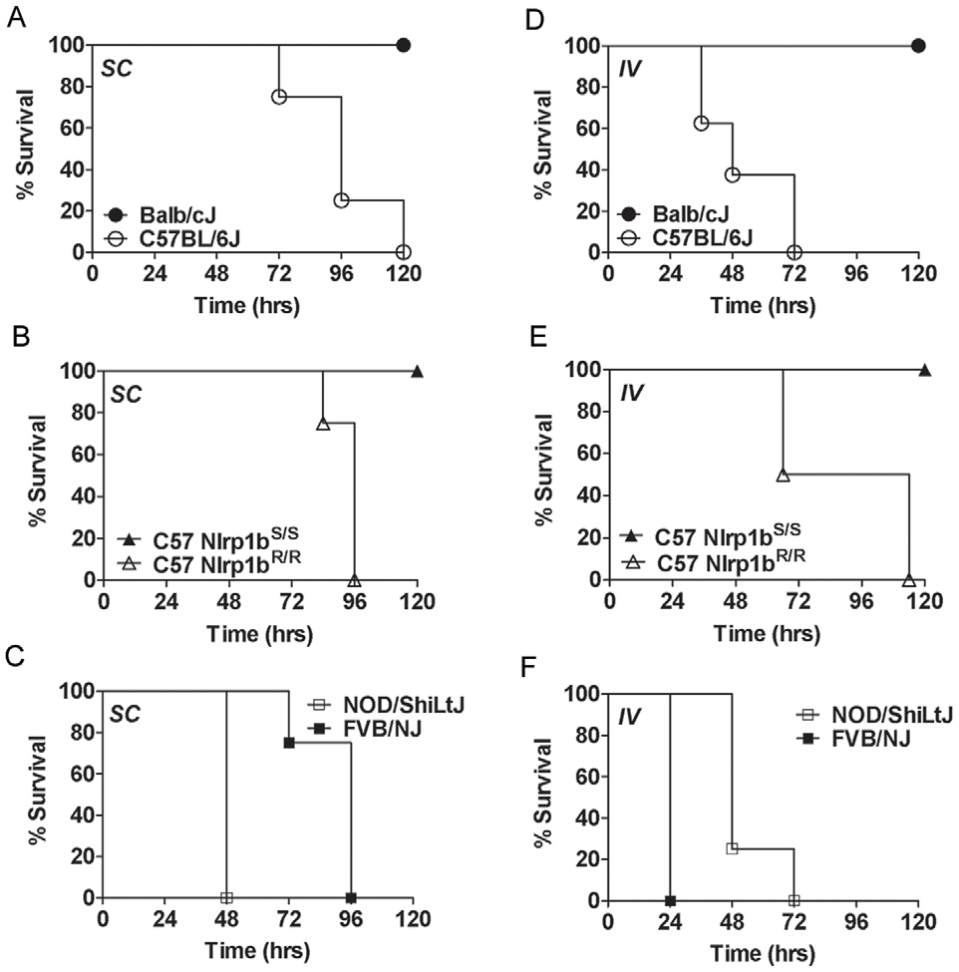
Susceptibility to infection with vegetative bacteria. Complement-sufficient Balb/cJ and C57BL/6J mice (panels A, D) or C57BL/6NTac *Nlrp1b^S/S^* and C57BL/6NTac *Nlrp1b^R/R^* mice (panels B, E) were challenged (1×10^5^ vegetative bacteria, 200 µl, SC (A, B) or IV (D, E)) and monitored for survival. Complement-deficient NOD/ShiLtJ and FVB/NJ mice were challenged with 1×10^4^ vegetative bacteria SC (C) or IV (F) and monitored. Open symbols denote mice harboring *Nlrp1b^S^* and closed circles are used for mice harboring *Nlrp1b^S^*. P-values for the comparison of the two curves in each panel are <0.01.

Further arguing against differences in germination playing any role in Nlrp1b-mediated control of infection was the finding that Nlrp1b^S^ also mediated resistance to SC infection with newly-germinated (i.e., vegetative) bacilli (Fig. 4A, 4B, 4C). To test whether Nlrp1b^S^ controls infection at the level of bacterial dissemination from the skin to other sites, we circumvented the dissemination step by directly introducing dividing vegetative bacteria into circulation. Strikingly, however, even the IV delivery of vegetative bacteria resulted in resistance of Nlrp1b^S^-expressing complement-sufficient mice relative to their counterpart Nlrp1b^R^-expressing mouse strains (Fig. 4D, 4E). Thus, Nlrp1b-mediated differences were not due to effects on bacterial spread from subcutaneous sites. In complement-deficient strains, however, Nlrp1b^S^-mediated resistance (tested here in FVB/NJ mice) was found only with SC infections (Fig. 4C, and data not shown). Nlrp1b^S^ did not impart resistance to these complement-deficient strains if the apparent Nlrp1b^S^-dependent inhibition of dissemination was overcome by introduction of vegetative bacteria into the bloodstream (Fig. 4F).

### Nlrp1b^S^-mediated resistance to infection requires caspase-1 and IL-1β

**Figure 5:**
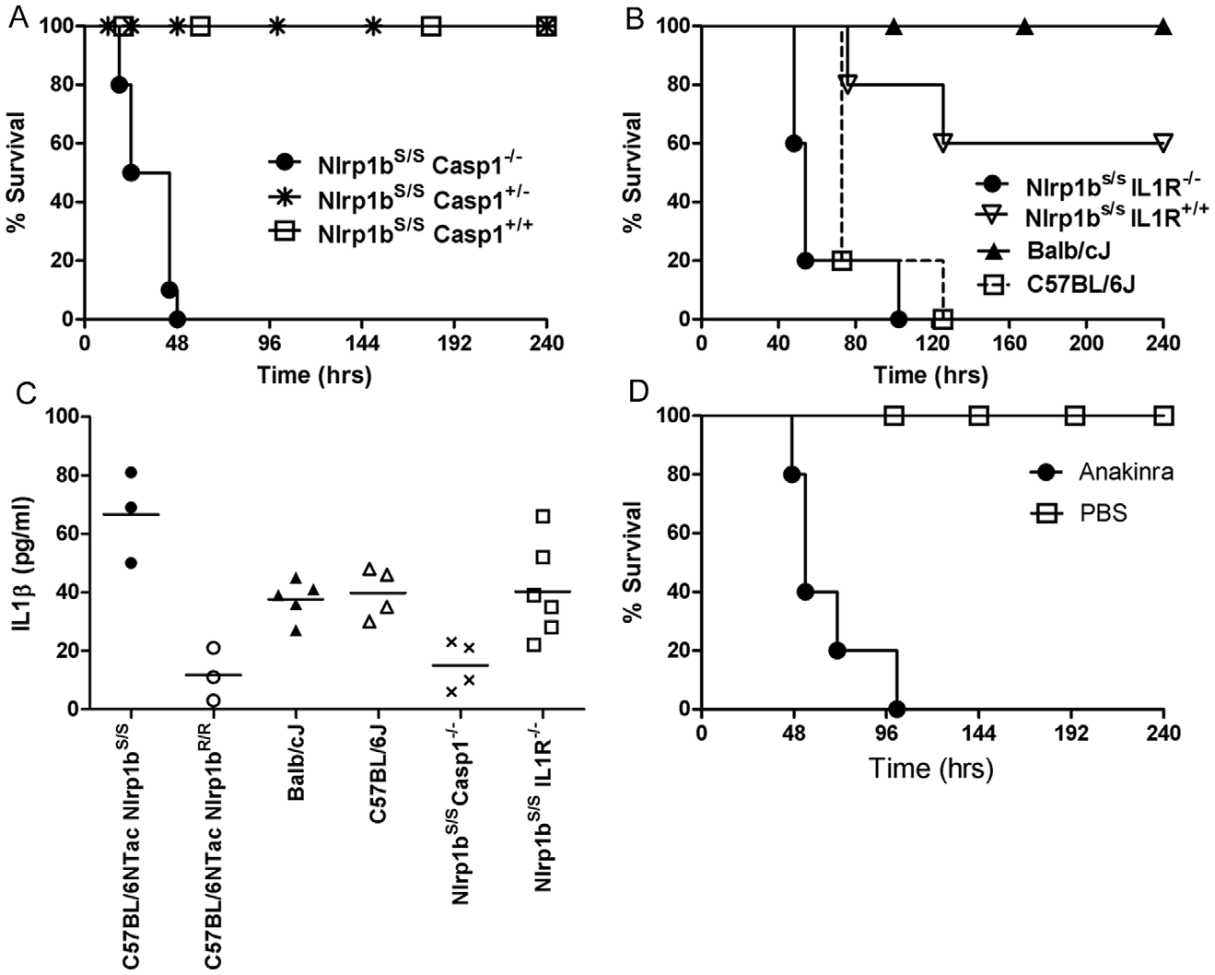
Nlrp1b^S^-mediated resistance to infection requires caspase-1 and IL-1β. (A) *Nlrp1b^S/S^Casp1^−/−^*, *Nlrp1b^S/S^Casp1^+/−^*, and *Nlrp1b^S/S^Casp1^+/+^* mice (Balb/cJ background, N4), were infected with 2×10^7^ spores (SC) and monitored for survival. Numbers per group were as follows: *Nlrp1b^S/S^ Casp1^−/−^* (n = 10), *Nlrp1b^S/S^ Casp1^+/−^* (n = 20), *Nlrp1b^S/S^ Casp1^+/+^* (n = 6)). P-values for the comparison of the *Nlrp1b^S/S^ Casp1^−/−^* to the three others strains are <0.0001. (B) Sensitivity of IL-1 receptor knockout mice (*Nlrp1b^S/S^IL1R^−/−^*) was compared to *Nlrp1b^S/S^IL1R^+/+^* ( = B6129SF2/J), Balb/cJ, and C57BL/6J mice (n = 5/group) following challenge with 2×10^7^ spores (SC). Curve comparisons: *Nlrp1b^S/S^IL1R^−/−^* vs. *Nlrp1b^S/S^IL1R^+/+^* (P = 0.0071); *Nlrp1b^S/S^IL1R^−/−^* vs. Balb/cJ (P = 0.0024). (C) Mice were infected with 5×10^7^ spores (SC) and IL-1β assessed in serum 6 h after infection. Each data point represents an individual mouse. P-value comparing C57BL/6NTac *Nlrp1b^S/S^* and C57BL/6NTac *Nlrp1b^R/R^* groups is 0.0062. P-value comparing Balb/cJ and C57BL/6J groups is 0.68. P-values comparing the *Nlrp1b^S/S^ Casp1^−/−^* to any other group except C57BL/6NTac *Nlrp1b^R/R^* are <0.03. (D) Balb/cJ mice (n = 5/group) were injected with 50 mg anakinra (IP) or PBS 1 h prior to spore infection, as well as 2, 6, 24, and 32 h post infection. Infection was with 6×10^7^ spores (SC). P-value comparing the two curves is 0.0022.

We previously observed that the earliest event associated with LT-mediated activation of Nlrp1b^S^ in *Nlrp1b^S/S^* mice is the appearance of IL-1β in circulation, occurring within 1.5–2.5 h after toxin injection [10], [11]. As caspase-1 and IL-1β have previously been associated with resistance to bacterial infections [24]–[26] and are also linked to macrophage killing of *B. anthracis* [27], we hypothesized that the LT-dependent IL-1β response in Nlrp1b^S^-expressing mice might explain their relative resistance. We generated *Nlrp1b^S/S^Casp1^−/−^* mice (in the Balb/cJ background, backcrossed four generations, N4). These mice harbor LT-resistant macrophages and do not produce an IL-1β burst in response to LT injection (data not shown). The mice were highly susceptible to spore infection when compared to their *Nlrp1b^S/S^Casp1^+/−^* and *Nlrp1b^S/S^Casp1^+/+^* (Balb/c backcrossed, N4) littermates (Fig. 5A), implicating caspase-1 in Nlrp1b^S^-mediated resistance to anthrax infection. IL-1 receptor knockout mice (*Nlrp1b^S/S^IL1R^−/−^*) were also tested against their wild type littermates (*Nlrp1b^S/S^IL1R^+/+^*) and found to be sensitive to spore infection (Fig. 5B). The macrophages from these mice, unlike those from *Nlrp1b^S/S^Casp1^−/−^* mice, were LT-sensitive and the mice produced an IL-1β burst in response to bolus LT injection in a manner similar to other Nlrp1b^S^-expressing mice (data not shown). However, the absence of IL-1 receptor prevented IL-1β signaling in these mice. The sensitivity of these mice to infection confirms that macrophage sensitivity to LT as a result of Nlrp1b status does not necessarily correlate with resistance to infection. Rather, it is likely the IL-1β generated in Nlrp1b^S^-expressing mice in response to LT is important to resistance. In fact, IL-1β responses measured at very early times after infection in various mice supported this hypothesis. C57BL/6NTac *Nlrp1b^S/S^* mice produced significantly higher levels of IL-1β than their C57BL/6NTac *Nlrp1b^R/R^* counterparts very early in infection, when toxin production is relatively low. Differences in IL-1β responses could not be discerned in the Balb/cJ and C57BL/6J mice at these early times, likely due to the many genetic differences which could influence their cytokine response to infection. Not surprisingly, there was little to no measurable response in *Nlrp1b^S/S^Casp1^−/−^* mice, while *Nlrp1b^S/S^IL1R^−/−^* mice had significant IL-1β production (Fig. 5C). Finally, resistant Nlrp1b^S^-expressing mice treated daily with the human IL-1 receptor antagonist anakinra (shown to be effective in rodents [28]–[30]), were sensitized to infection (Fig. 5D), confirming a role for IL-1 signaling in resistance to infection.

### Nlrp1b^S^, caspase-1, and IL-1 act to control infection by enhancing neutrophil responses

**Figure 6:**
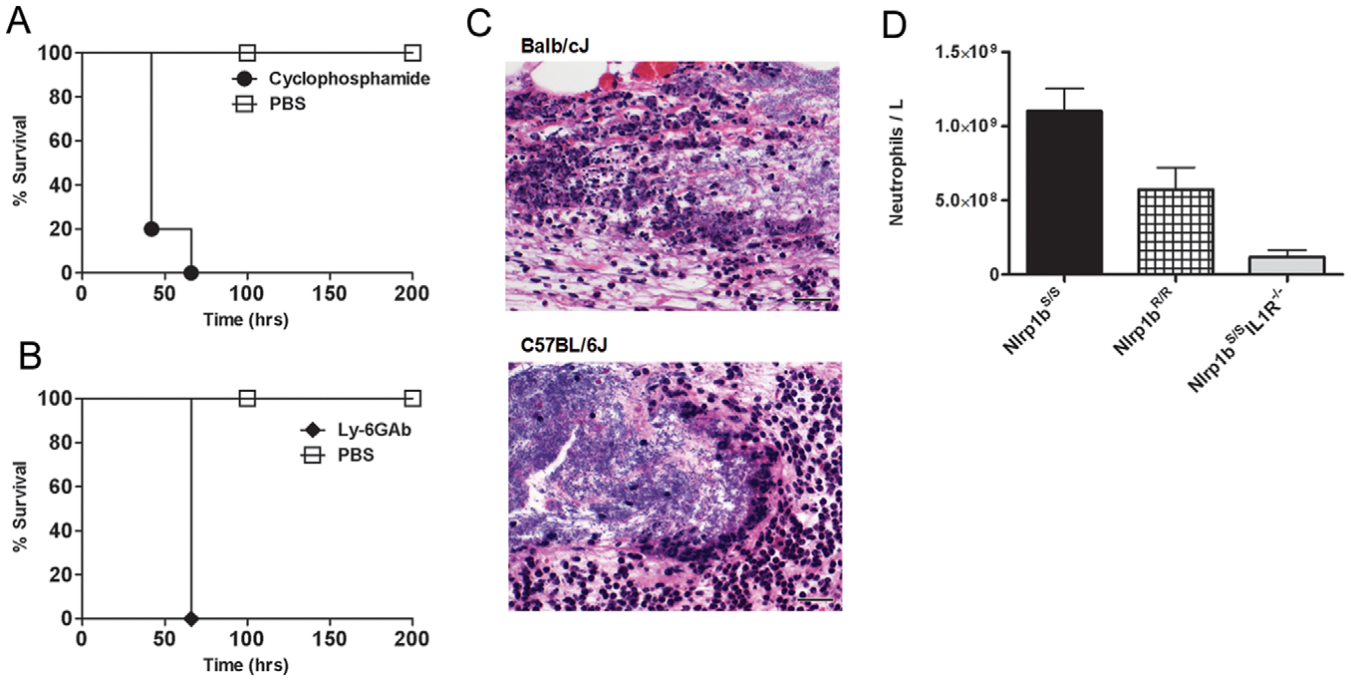
Neutrophils protect against *B. anthracis* infection. Balb/cJ mice were treated with cyclophosphamide (A) or a neutrophil-depleting antibody (B) prior to SC challenge with the dose of 1×10^7^ spores (to which they are normally resistant) and compared to PBS-treated controls receiving the same spore challenge. P-values for comparison of the two curves in each panel are <0.003. (C) H&E stain of infected skin sections taken 8 h after infection of Balb/cJ and C57BL/6J mice. Left panels represent images taken at 50× (scale bar = 200 micron); right panels are higher magnification (400×) detail of a region from left panels showing the boundaries of bacteria and neutrophils (scale bar = 25 microns). (D) C57BL/6NTac *Nlrp1b^S/S^* and C57BL/6NTac *Nlrp1b^R/R^* and *Nlrp1b^S/S^IL1R^−/−^* mice were infected with 6×10^7^ spores (SC) and cell blood counts analyzed after 4 h. The neutrophil counts shown are for n = 4 mice/group. Baseline neutrophil counts for uninfected mice were an average of 4.45×10^8^/ml. P-values comparing any two groups are <0.05.

We previously showed that LT injection in Balb/cJ mice induces an Nlrp1b^S^-dependent IL-1β release, the subsequent induction of numerous cytokines and chemokines [10], [11], including the potent neutrophil chemokine KC (mouse IL-8), leading to a massive increase in circulating neutrophils [11]. A similar response was absent in Nlrp1b^R^-expressing mice [10], [11]. To confirm the importance of neutrophils in clearance of *B. anthracis* bacteria, Balb/cJ (*Nlrp1b^S/S^*) mice were depleted of neutrophils by two different methods. Pre-treatment with cyclophosphamide or anti-Ly-6G monoclonal antibody 1A8 [31] sensitized Balb/cJ mice to a spore dose to which they are normally resistant (Fig. 6A, 6B). The protective role of neutrophils was also observed in infection trials in C57BL/6J, A/J, and SWR/J mice (all harboring *Nlrp1b^R/R^*), as they were similarly sensitized following neutrophil depletion (data not shown). Histological analyses of spore-infected Balb/cJ, C57BL/6NTac *Nlrp1b^S/S^*, C57BL/6NTac *Nlrp1b^R/R^* and C57BL/6J animals showed similar destruction of neutrophils at the boundaries of bacteria-neutrophil interaction in SC sections for both mouse strains (Fig. 6C and data not shown). Analysis of circulating neutrophil levels, however, showed significantly higher numbers of neutrophils in Nlrp1b^S^-expressing mice (Fig. 6D) as early as 4 h following SC spore infection, in a manner paralleling the higher numbers of circulating neutrophils in Balb/cJ mice when compared to C57BL/6J mice after bolus LT challenge [11]. Furthermore, significantly lower circulating neutrophil numbers were also associated with spore-sensitive *Nlrp1b^S/S^ IL1R^−/−^* mice, likely due to their inability to respond to IL-1β (Fig. 6D). Thus, it appears that Nlrp1b^S^ expressing mice may have a stronger neutrophilic response to infection following the germination of *B. anthracis* and toxin production, accounting for the resistance associated with Nlrp1b status. Interestingly, we found that CXCR2 (KC, IL-8 receptor) knockout mice in either *Nlrp1b^R/R^* or *Nlrp1b^S/S^* backgrounds did not show differences in susceptibility to SC spore infection from wild type littermate controls (data not shown). Furthermore, administration of a KC (IL-8) neutralizing antibody did not sensitize Balb/cJ mice to spore infection (data not shown). Thus, IL-1β mediated control of neutrophil recruitment does not appear to occur via the strong KC response that is induced by LT strictly in *Nlrp1b^S/S^* strains.

## Discussion

We previously used anthrax toxin receptor knockout mice to demonstrate that anthrax toxin is essential for bacterial dissemination in the complement-sufficient C57BL/6 (*Nlrp1b^R/R^*) background [13]. These results complemented findings in an aerosol murine model using complement-deficient A/J mice (*Nlrp1b^R/R^*) infected with spores of Sterne strain mutants which were deleted for individual toxin components [12]. Thus, in both C57BL/6J and A/J strains (both Nlrp1b^R^-expressors and harboring LT-resistant macrophages), bacterial subversion of the host innate immune response depends on LT (produced by the bacterium) and host cell PA receptors (to allow LF to enter cells). These earlier findings fit well with the general consensus that LT impairment of the innate immune response in a variety of cells aids in establishing disease at early stages of *B. anthracis* infection (for review, see [32]).

The role of toxin in spore infection in Nlrp1b^S^-expressing mice harboring LT-sensitive macrophages appears to be quite different. Data presented here indicate that in these animals the role of toxin is likely defined by a balance between its inhibition of immune cell function through targeting the MEK-pathways (which also occurs in Nlrp1b^R^-expressing mice) and the consequences of *Nlrp1b* ^S^ status in macrophages and/or other cell types. Our findings indicate that the *Nlrp1b^S^* allele confers relative resistance to spore infection, supporting findings reported using Nlrp1b transgenic mice [23]. Furthermore, we find that Nlrp1b-mediated resistance requires both caspase-1 and IL-1β signaling.

We tested several hypotheses regarding mechanisms by which Nlrp1b^S^ might impart resistance to infection. Nlrp1b status did not affect spore germination, which occurred rapidly following SC administration of spores and was observed in SC tissues that appeared largely devoid of macrophages. This finding was confirmatory of previous reports showing spore germination in SC infection sites by 2 h after inoculation in both CBA/J (*Nlrp1b^S/S^*) and A/J (*Nlrp1b^R/R^*) mice in a manner independent of complement status [33]. Nlrp1b^S^-mediated resistance to both SC and IV infections with vegetative bacteria confirmed that Nlrp1b^S^ could manifest its control of infections at steps subsequent to germination of spores and dissemination of bacteria.

We hypothesize that Nlrp1b^S^-mediated IL-1β release and its associated cytokine and chemokine burst [10], [11] may be the initiating events providing a protective response against spore infection in a complement-independent manner. IL-1β release requires caspase-1 activity and its action requires functional IL-1 receptors. *Nlrp1b^S/S^Casp1^−/−^* mice which harbored LT-resistant macrophages and did not activate and release IL-1β in response to LT injection were fully susceptible to infection when compared to *Nlrp1b^S/S^ Casp1^+/−^* and *Nlrp1b^S/S^ Casp1^+/+^* littermates, confirming that caspase-1 was required for Nlrp1b^S^-mediated resistance to anthrax infection. A similar result was obtained in a caspase-1 knockout mouse in the C3H/HeN (*Nlrp1b^S/S^*) background, when infection was by the intraperitoneal (IP) route [27]. IL-1 receptor knockout mice harboring *Nlrp1b^S/S^* alleles (*Nlrp1b^S/S^IL1R^−/−^*) were also susceptible to spore infection despite having LT-sensitive macrophages, activating caspase-1 and producing an IL-1β response to LT. The sensitivity of these mice suggests that IL-1β signaling activity is required for Nlrp1b^S^-mediated resistance and that macrophage sensitivity to LT is not the determining factor in infection outcome. Rather, the consequences of macrophage lysis (i.e., rapid IL-1β release) may be the more important factor determining resistance. Sensitization of resistant Nlrp1b^S^-expressing mice following treatment with an IL-1 receptor antagonist further confirms the importance of IL-1β signaling in mediating Nlrp1b effects.

One mechanism through which Nlrp1b^S^-dependent IL-1β responses could control bacterial clearance is IL-1β mediated neutrophil recruitment. Previous reports demonstrated a role for neutrophils in control of Sterne infection in mice [33]–[36] and the ability of human neutrophils to kill *B. anthracis* [37]. Data supporting a potential role for neutrophils in controlling infection in Nlrp1b^S^-expressing mice came from prior studies showing the induction of neutrophil chemokines accompanied by strikingly high levels of circulating neutrophils in LT-treated Balb/cJ (but not C57BL/6J) mice [11]. In the current study, we found significantly higher levels of neutrophils in Nlrp1b^S^-expressing mice compared to Nlrp1b^R^-expressing mice following infection. These findings emphasize the role of Nlrp1b^ S^ in differential recruitment of neutrophils in response to anthrax infection. The observation that neutrophil depletion by either chemical or biological methods leads to a significant sensitization of four different mouse strains to anthrax infection further confirms the role this cell type plays in resistance against *B. anthracis* infection.

The results in our current study point to the complexities associated with the use of rodents as models for *B. anthracis* infection. The wide range of susceptibilities associated with complement status and the *Nlrp1b*-dependent control of infection are described at length here. Human macrophages tested (to date) are resistant to LT lysis (data not shown). Thus, complement-sufficient *Nlrp1^R^* mouse strains such as C57BL/6J may be the best available mouse model for study of the response of human cells. It would be interesting to search a range of animal species for the presence of *Nlrp1b^S^* alleles that confer protection against infection, like the *Nlrp1b^S^* alleles described here in mice. This could help to determine whether evolutionary pressure has led to maintenance of the *Nlrp1b^S^* allele in these species.

## Materials and Methods

### Ethics Statement

All animal experiments were performed in strict accordance with guidelines from the NIH and the Animal Welfare Act, approved by the Animal Care and Use Committee of the National Institute of Allergy and Infectious Diseases, National Institutes of Health.

### Bacteria

*S*pores were prepared from the nonencapsulated, toxigenic *B. anthracis* Ames 35 (A35) strain [38] as previously described [39]. Briefly, bacteria were grown on sporulation agar at 37°C for 3 days followed by 4 days at 4°C, and were inspected by microscopy to verify >95% sporulation. Spores were purified from plates by four rounds of centrifugation and sterile water washes, followed by heat treatment at 70°C for 30 min (to kill any vegetative bacteria). For studies using vegetative bacteria, spores were grown in LB broth and diluted in PBS prior to injection. Vegetative bacteria were quantified by adsorption readings, with an A_600_ of 1.0 corresponding to a concentration of 2×10^7^ CFU/ml, as verified by dilution plating. Spore quantification was performed using a Petroff Hausser counting chamber (Hausser Scientific, Horsham, PA) and verified by dilution plating.

### Materials

Materials obtained from commercial sources included cyclophosphamide (Baxter Healthcare, Deerfield, IL), neutralizing anti-KC antibody (R&D Systems, Minneapolis, MN), anti-Ly-6G antibody 1A8 (BD Pharmingen, San Diego, CA) and anakinra (Kineret, Amgen, Thousand Oaks, CA).

### Animals

All inbred mice were obtained from Jackson Laboratories (Bar Harbor, Maine). IL-1 receptor knockout mice on a 129B6SF/2J background (Jackson Laboratory B6;129S1-IL1r1^tm1Roml^/J mice) were genotyped and mice harboring Nlrp1b^S/S^ alleles were bred in our facility to establish the *Nlrp1b^S/S^IL1R^−/−^* line. C57BL/6NTac *Nlrp1b^S/S^* and C57BL/6NTac *Nlrp1b^R/R^* were generated from mice that were previously described [10]. The parents for these mice were generated through 7 backcrosses of previously described C57Bl/6Ai-[KO]*NOS2* mice homozygous for either the *Nlrp1b^S^* or *Nlrp1b^R^* allele on the C57BL/6NTac background, and are identical at >98% of loci. CXCR2^−/−^ mice were generated at the NIH by Drs. Ji-Liang Gao and Christophe Cataisson. We created a complement-sufficient caspase-1 knockout mouse homozygous for the *Nlrp1b^S^* allele on the Balb/cJ background through backcrossing NOD.129S2(B6)- *Casp1^tm1Sesh^*/LtJ mice (Jackson Laboratories)(*Nlrp1b^R/R^)* with Balb/cJ mice (*Nlrp1b^S/S^)*, followed by breeding of the F1 generation sibling mice (*Casp1^+/−^ X Nlrp1b^R/S^*). *Nlrp1b^S/S^Casp1^−/−^* progeny were backcrossed to Balb/cJ in this manner for four generations.

### Genotyping

*Nlrp1b* genotype was determined by PCR (using the forward primer: 5′- AGG CAA GCA CAT TTG ACT TCA AGG T-3′ and the reverse primer 5′-GCT CAG TAG CTG CAT CTT GCA TTT C-3′) followed by XhoI digestion. Caspase-1 genotyping was by PCR. Wild type caspase-1 alleles were detected by wild type-specific primer 5′-GCA TGC CTG AAT AAT GAT CAC C-3′ and a common primer 5′- GAA GAG ATG TTA CAG AAG CC-3′. The caspase-1 deleted allele was detected by the amplification of a fragment in the presence of a caspase-1 deletion specific primer (5′-GCG CCT CCC CTA CCC GG-3′) and the common primer.

### Infection, dissemination, and edema studies

Mice were injected subcutaneously (SC, 200 µl) in the scruff of the neck with various doses of spores prepared in PBS. Alternatively, vegetative *B. anthracis* bacteria suspended in PBS were injected SC (200 µl) or IV (200 µl). Infected animals were monitored three times daily for 10 days for signs of malaise or mortality. For IL-1β analyses, mice were bled and serum separator tubes (Sarstedt, Newton, NC) were used to separate serum for cytokine measurements. For cell blood count (CBC) analyses, mice were bled by cardiac puncture directly into EDTA (5 mM)-coated syringes and EDTA-coated blood collection tubes (Sarstedt, Newton, NC). For neutrophil depletion by cyclophosphamide, mice were treated with the drug IP (100 mg/kg, prepared in 500 µl PBS, 72 and 48 h prior to infection, and 200 mg/kg 24 h prior to infection). For neutrophil depletion using antibody 1A8 (anti-Ly-6G), 100 µg of antibody prepared in PBS was injected twice (IP), at 24 h and 6 h prior to spore infection of mice. For KC neutralization, 100 µg of antibody prepared in PBS was injected SC at the spore injection site 30 min prior to spore infection. For anakinra studies, drug was diluted in PBS and injected IP (50 mg/20 g mouse) 1 h prior to infection, as well as 2, 6, 24, and 32 h post infection. In dissemination studies, organs (spleen, liver and both kidneys) were collected from infected animals at 24 h after infection and pooled prior to homogenization using a Tissue Tearor Homogenizer (BioSpec Products, Oklahoma). Homogenates were adjusted to the same volume (5 ml) in sterile PBS prior to dilution and plating on LB agar for CFU assessment. Lung preparations were made separately and processed similarly. In experiments assessing leg edema following infection, 2×10^7^ spores were injected SC in a specific location of each right foreleg in a 50 µl volume. This site was chosen to limit the ability of edema spread, thus allowing consistent quantification of edema. Edema was assessed at 1, 2, 4, 8, and 24 h after infection by measuring the horizontal anterior/posterior and sagittal dorsal/ventral directions using digital calipers (Mitutoyo Corporation, Aurora, IL).

### Histological analyses

Skin and underlying edematous tissue sections were taken following SC spore infection and fixed overnight in 10% formalin followed by repeated 70% ethanol washes. Section preparation and hematoxylin and eosin (H&E) staining were performed by Histoserv, Inc. (Gaithersburg, MD).

### IL-1β responses to lethal toxin and macrophage sensitivity tests

Mice were injected with LF and PA purified as previously described [40] and prepared in sterile PBS. The LF used here is a recombinant protein having an N-terminal sequence beginning HMAGG. Mice were injected IP with 100 µg of LT (100 µg PA plus 100 µg LF). IL-1β analyses were performed on serum bleeds by ELISA as previously described [11]. LT toxicity studies on bone marrow macrophages were performed as previously described [8].
